# Supplementary material for: Accuracy of digital chest x-ray analysis with artificial intelligence software as a triage and screening tool in hospitalized patients being evaluated for tuberculosis in Lima, Peru
Source: PLOS Glob Public Health. 2024 Feb 7;4(2):e0002031. doi: 10.1371/journal.pgph.0002031 (PMC10849246; doi:10.1371/journal.pgph.0002031)
Supplement: S1 Table — a: Summary of Culture and Xpert results concordance. b: Xpert sensitivity for smear-positive and smear-negative culture-positive TB in triage cohort patients. (DOCX) [file pgph.0002031.s002.docx]

**S1 Table**

Table S1a: Summary of Culture and Xpert result concordance.

|  | Culture Negative  (n=322) | Culture  Positive  (n=65) | Missing  Culture  (n=52) |
| --- | --- | --- | --- |
| Xpert Negative  (n=329) | 298 | 4 | 27 |
| Xpert Positive  (n=69) | 9 | 55 | 5 |
| Missing Xpert  (n=41) | 15 | 6 | 20 |

Table S1b: Xpert sensitivity for smear-positive and smear-negative culture-positive TB in triage cohort patients

|  | Sensitivity – smear positive culture positive  (95% CI) | Sensitivity – smear negative  culture positive  (95% CI) |
| --- | --- | --- |
| Xpert | (35/37)  94.6%  (81.8-99.3%) | (12/14)  85.7%  (57.2-98.2%) |
