# Supplementary material for: Accuracy of digital chest x-ray analysis with artificial intelligence software as a triage and screening tool in hospitalized patients being evaluated for tuberculosis in Lima, Peru
Source: PLOS Glob Public Health. 2024 Feb 7;4(2):e0002031. doi: 10.1371/journal.pgph.0002031 (PMC10849246; doi:10.1371/journal.pgph.0002031)
Supplement: S2 Table — (DOCX) [file pgph.0002031.s003.docx]

**Table S2: Demographic and clinical characteristics of enrolled participants compared to those who were excluded.**

|  | Triage (n=419) | Screening (n=184) | Excluded-  Triage (n=475) | P-value* | Excluded- Screening  (n=24) | P-value** |
| --- | --- | --- | --- | --- | --- | --- |
| **Median Age (years, interquartile range)** | 41.35 (26.8, 56.6) | 36.19 (25.19, 50.53) | 37.33 (26.2, 51.8) | **0.028** | 35.27 (24.7, 45.6) | 0.632 |
| **Sex, No (%)**  Female  Male | 164 (39.1)  255 (60.9) | 111 (60.3)  73 (39.7) | 246 (51.8)  229 (48.2) | **<0.001** | 14 (58.3)  10 (41.7) | 1.000 |
| **History of Previous TB, No (%)**  Yes  No  Refused | 140 (33.4)  278 (66.4)  1 (0.20) | 0 (0.00)  184 (100)  0 (0.00) | 158 (33.3)  317 (66.7)  0 (0.00) | 0.771 | 0 (0.00)  24 (100)  0 (0.00) | - |
| **HIV, No (%)**  Yes  No | 36 (8.6)  383 (91.4) | 1 (0.5)  183 (99.5) | 29 (6.1)  446 (93.9) | 0.158 | 1 (4.2)  23 (95.8) | 0.218 |
| **Smoking, No (%)**  Never  Former  Current | 202 (48.2)  161 (38.4)  56 (13.4) | 99 (53.8)  51 (27.7)  34 (18.5) | 292 (61.5)  133 (28.0)  50 (10.5) | **<0.001** | 13 (54.2)  6 (25.0)  5 (20.8) | 0.944 |
| **Alcohol, No (%)**  Never  Former  Current  Missing | 107 (25.5)  121 (28.9)  189 (45.1)  2 (0.5) | 32 (17.3)  33 (18.0)  119 (64.7)  0 (0.00) | 125 (26.3)  105 (22.1)  244 (51.4)  1 (0.2) | 0.242 | 4 (16.7)  7 (29.2)  13 (54.2)  0 (0.00) | 0.433 |
| **Respiratory Disease, No (%)**  Asthma  Bronchiectasis  None | 28 (6.7)  13 (3.1)  378 (90.2) | 2 (1.1)  0 (0.00)  182 (98.9) | 22 (4.6)  17 (3.6)  436 (91.8) | 0.390 | 0 (0.00)  0 (0.00)  24 (100) | 0.608 |
| **Diabetes, Type II, No (%)**  Yes  No | 58 (13.8)  361 (86.2) | 25 (13.6)  159 (86.4) | 44 (9.3)  431 (90.7) | **0.035** | 2 (8.3)  22 (91.7) | 0.747 |
| **Prison, No (%)**  Yes  No | 62 (14.8)  357 (85.2) | 3 (1.6)  181 (98.4) | 41 (8.6)  434 (91.4) | **0.005** | 1 (4.2)  23 (95.8) | 0.390 |
| **Household Contact of TB positive patient, No (%)**  Yes  No  Missing | 159 (38.0)  256 (61.1)  4 (0.9) | - | 152 (32.0)  317 (66.7)  6 (1.3) | 0.165 | 3 (12.5)  21 (87.5)  0 (0.00) | - |
| **Smear Status, No (%)**  Positive  Negative  Missing | 48 (11.5)  363 (86.6)  8 (1.9) | 0 (0.00)  183 (99.5)  1 (0.5) | 54 (11.4)  411 (86.5)  11 (2.3) | 0.420 | 0 (0.00)  19 (79.2)  5 (20.8) | 1.000 |
| **Cough, No (%)**  *Length, in Weeks*  Less than 1 week  1-2 weeks  More than 2 weeks  Missing  *Phlegm*  Yes  No  Missing  *Blood*  Yes  No  Missing | 102 (24.4)  107 (25.5)  189 (45.1)  21 (5.0)  352 (84.0)  47 (11.2)  20 (4.8)  166 (39.6)  233 (55.6)  20 (4.8) | -  -  - | 148 (31.2)  97 (20.4)  189 (39.8)  41 (8.6)  373 (78.5)  64 (13.5)  38 (8.0)  135 (28.4)  302 (63.6)  38 (8.0) | 0.065  0.071  **0.001** | -  -  - | -  -  - |
| **Fever, No (%)**  Yes  No  Refused | 265 (63.3)  153 (36.5)  1 (0.2) | 85 (46.2)  99 (53.8)  0 (0) | 248 (52.2)  227 (47.8)  0 (0.0) | **0.001** | 7 (29.2)  17 (70.8)  0 (0.00) | 0.130 |
| **Night Sweats in the last 3 months, No (%)**  Yes  No  Refused | 251 (59.9)  168 (40.1)  0 (0.0) | 52 (28.3)  132 (71.7)  0 (0.0) | 248 (52.2)  226 (47.6)  1 (0.2) | **0.026** | 5 (20.8)  19 (79.2)  0 (0.0) | 0.627 |
| **Weight Loss (unintentional), No (%)**  Yes  No  Refused | 293 (69.9)  123 (29.4)  3 (0.7) | 84 (45.6)  98 (53.3)  2 (1.1) | 293 (61.7)  182 (38.3)  0 (0.0) | **0.002** | 10 (41.6)  13 (54.2)  1 (4.2) | 0.349 |
| **Difficulty Breathing, No (%)**  Yes  No | 335 (80.0)  84 (20.0) | 52 (28.3)  132 (71.7) | 310 (65.3)  165 (34.7) | **<0.001** | 6 (25.0)  18 (75.0) | 0.814 |

*Triage vs Excluded from triage cohort

**Screening vs Excluded from screening cohort
