# Supplementary material for: Accuracy of digital chest x-ray analysis with artificial intelligence software as a triage and screening tool in hospitalized patients being evaluated for tuberculosis in Lima, Peru
Source: PLOS Glob Public Health. 2024 Feb 7;4(2):e0002031. doi: 10.1371/journal.pgph.0002031 (PMC10849246; doi:10.1371/journal.pgph.0002031)
Supplement: S3 Table — (DOCX) [file pgph.0002031.s004.docx]

**Table S3: Diagnostic accuracy of qXR Version 3 and 4 using a reference standard which is positive if either mycobacterial culture or Xpert is positive for the triage cohort.**

|  | Sensitivity  (95% CI) | Specificity  (95% CI) | AUC  (95% CI) |
| --- | --- | --- | --- |
| qXR Version 3 | | | |
| Manufacturer Threshold 0.5 | (73/79)  92.4%  (84.2-97.2%) | (96/298)  32.2%  (26.9-37.8%) | 0.778  (0.719-0.837) |
| qXR Version 4 | | | |
| Manufacturer Threshold 0.5 | (73/79)  92.4%  (84.2-97.2%) | (97/298)  32.6%  (27.3-38.2%) | 0.775  (0.716-0.834) |
