# Supplementary material for: Accuracy of digital chest x-ray analysis with artificial intelligence software as a triage and screening tool in hospitalized patients being evaluated for tuberculosis in Lima, Peru
Source: PLOS Glob Public Health. 2024 Feb 7;4(2):e0002031. doi: 10.1371/journal.pgph.0002031 (PMC10849246; doi:10.1371/journal.pgph.0002031)
Supplement: S4 Table — (DOCX) [file pgph.0002031.s005.docx]

**Table S4: Summary of Diagnostic Accuracy for qXR version 3 compared to the culture (primary) and Xpert (secondary) reference standards**

|  | Triage Patients | | | Screening Patients | | |
| --- | --- | --- | --- | --- | --- | --- |
|  | Sensitivity  (95% CI) | Specificity  (95% CI) | AUC  (95% CI) | Sensitivity  (95% CI) | Specificity  (95% CI) | AUC  (95% CI) |
| Culture | | | | | | |
| qXR Version 3 | | | | | | |
| Manufacturer Threshold 0.5 | 90.8%  59/65  (81-96.5%) | 31.7%  102/322  (26.6-37.1%) | 0.780  (0.717, 0.843) | ^ | 94.2%  162/172  (89.5-96.9%) | - |
| Threshold 0.675* | 90.8%  59/65  (81-96.5%) | 41.3%  133/320  (35.9-46.9%) | - | ^ | 97.1%  167/172  (93.2-98.8%) | - |
| Xpert | | | | | | |

| qXR Version 3 | | | | | | |
| --- | --- | --- | --- | --- | --- | --- |
| Manufacturer Threshold 0.5 | 92.8%  64/69  (83.9-97.6%) | 31.9%  105/329  (26.9-37.3%) | 0.759  (0.696, 0.821) | 100%  1/1  (2.5-100%) | 94.4%  169/179  (90.0-97.3%) | 1.00  (-, 1.00) |
| Threshold 0.6* | 89.9%  62/69  (80.2-95.8%) | 36.8%  121/329  (31.6-42.2%) | - | 100%  1/1  (2.5-100%) | 97.2%  174/179  (93.6-99.1%) | - |

*threshold at which sensitivity is closest to 90%

^No positive cultures in the Screening Group
