# Supplementary material for: Accuracy of digital chest x-ray analysis with artificial intelligence software as a triage and screening tool in hospitalized patients being evaluated for tuberculosis in Lima, Peru
Source: PLOS Glob Public Health. 2024 Feb 7;4(2):e0002031. doi: 10.1371/journal.pgph.0002031 (PMC10849246; doi:10.1371/journal.pgph.0002031)
Supplement: S5 Table — (DOCX) [file pgph.0002031.s006.docx]

**Table S5: Diagnostic accuracy of qXR Version 3 and 4 for pre-specified subgroups in the triage cohort for which participants with prior TB, respiratory diseases, and HIV, were excluded**

|  | Excluding Patients with Prior TB | | | Excluding Patients with Respiratory Diseases | | | Excluding People with HIV | | |
| --- | --- | --- | --- | --- | --- | --- | --- | --- | --- |
| Threshold | Sensitivity  (95% CI) | Specificity  (95% CI) | AUC  (95% CI) | Sensitivity  (95% CI) | Specificity  (95% CI) | AUC  (95% CI) | Sensitivity  (95% CI) | Specificity  (95% CI) | AUC (95% CI) |
| Culture | | | | | | | | | |
| qXR Version 3 | | | | | | | | | |
| Manufacturer Threshold 0.5 | (46/52)  88.5% (76.6-95.6%) | (84/208)  40.4% (33.7-47.4%) | 0.800 (0.730, 0.870) | (58/64)  90.6% (80.7-96.5%) | (92/285)  32.3% (26.9-38.0%) | 0.784 (0.719, 0.848) | (54/59)  91.5% (81.3-97.2%) | (89/294)  30.3% (25.1-35.9%) | 0.792 (0.730, 0.854) |
| qXR Version 4 | | | | | | | | | |
| Manufacturer Threshold 0.5 | (46/52)  88.5% (76.6-95.6%) | (84/208)  40.4%  (33.7-47.4%) | 0.802 (0.731, 0.872) | (58/64)  90.6% (80.7-96.5%) | (93/285)  32.6% (27.2-38.4%) | 0.781 (0.716, 0.846) | (54/58)  93.1% (83.3-98.1%) | (90/294)  30.6%  (25.4-36.2%) | 0.789 (0.726, 0.851) |
| Xpert | | | | | | | | | |
| qXR Version 3 | | | | | | | | | |
| Manufacturer Threshold 0.5 | (49/54)  90.7%  (79.7-96.9%) | (87/213)  40.8% (34.2-47.8%) | 0.784 (0.715, 0.854) | (63/68)  92.6% (83.7-97.6%) | (94/292)  32.2% (26.9-37.9%) | 0.763 (0.698, 0.827) | (58/61)  95.1% (86.3-99%) | (92/302)  30.5% (25.3-36%) | 0.778 (0.716, 0.840) |
| qXR Version 4 | | | | | | | | | |
| Manufacturer Threshold 0.5 | (49/54)  90.7% (79.7-96.9%) | (86/213)  40.4%  (33.7-47.3%) | 0.784 (0.714, 0.854) | (63/68)  92.6% (83.7-97.6%) | (95/292)  32.5% (27.2-38.2%) | 0.758 (0.693, 0.823) | (58/61)  95.1% (86.3-99%) | (93/302)  30.8% (25.6-36.3%) | 0.775 (0.712, 0.837) |
