# Supplementary material for: Accuracy of digital chest x-ray analysis with artificial intelligence software as a triage and screening tool in hospitalized patients being evaluated for tuberculosis in Lima, Peru
Source: PLOS Glob Public Health. 2024 Feb 7;4(2):e0002031. doi: 10.1371/journal.pgph.0002031 (PMC10849246; doi:10.1371/journal.pgph.0002031)
Supplement: S6 Table — (DOCX) [file pgph.0002031.s007.docx]

**Table S6: Lung abnormalities detected by qXR analysis for the Triage and Screening cohorts**

|  | **Triage**  (N=419)  n/N, %  (95% CI) | **Screening**  (N=184)  n/N, %  (95% CI) |
| --- | --- | --- |
|  |  |  |
| Atelectasis | 93/419  22.2%  (18.4-26.4%) | 14/184  7.61%  (4.5-12.5%) |
| Blunted CP | 17/419  4.06%  (2.5-6.4%) | 5/184  2.72%  (1.1-6.4%) |
| Calcification | 68/419  16.2%  (13.0-20.1%) | 2/184  1.09%  (0.3-4.3%) |
| Cardiomegaly | 36/419  8.59%  (6.3-11.7%) | 17/184  9.24%  (5.8-14.4%) |
| Cavity | 80/419  19.1%  (15.6-23.2%) | 0/184  0%  (0-0%) |
| Consolidation | 261/419  62.3%  (57.5-66.8%) | 7/184  3.80%  (1.8-7.8%) |
| Fibrosis | 195/419  46.5%  (41.8-51.3%) | 19/184  10.3%  (6.7-15.7%) |
| Hilar Lymphadenopathy | 2/419  0.48%  (0.1-1.9%) | 0/184  0%  (0-0%) |
| Nodule | 242/419  57.8%  (53.0-62.4%) | 20/184  10.9%  (7.1-16.3%) |
| Opacity | 341/419  81.4%  (77.4-84.8%) | 44/184  23.9%  (18.3-30.7%) |
| Pleural Effusion | 151/419  36.0%  (31.6-40.8%) | 9/184  4.89%  (2.6-9.2%) |
