# Supplementary material for: Accuracy of digital chest x-ray analysis with artificial intelligence software as a triage and screening tool in hospitalized patients being evaluated for tuberculosis in Lima, Peru
Source: PLOS Glob Public Health. 2024 Feb 7;4(2):e0002031. doi: 10.1371/journal.pgph.0002031 (PMC10849246; doi:10.1371/journal.pgph.0002031)
Supplement: S1 Text — (DOCX) [file pgph.0002031.s010.docx]

**SUPPLEMENT_S1 Text**

*qXR version 3*

Using the culture reference standard for pulmonary tuberculosis, qXR v3 (at the manufacturer pre-specified threshold of 0.5) had an overall sensitivity of 0.91 (59/65, 95% CI 0.81-0.97), specificity of 0.32 (102/322, 95% CI 0.27-0.37), and AUC of 0.78 (95% CI 0.72-0.84) (Table S5). Using the Xpert reference standard for pulmonary tuberculosis, qXR v3 (at the manufacturer pre-specified threshold of 0.5) had an overall sensitivity of 0.93 (64/69, 95% CI 0.84-0.98), specificity of 0.32 (105/329, 95% CI 0.31-0.41), and AUC of 0.76 (95% CI 0.70-0.82) (Table S5). When sensitivity was set at 90% to match the WHO triage test accuracy performance criteria, specificity rose to 0.41 (133/320, 95% CI 0.36-0.47) and 0.37 (121/329, 95% CI 0.32-0.42) with the culture and Xpert reference standards respectively (S5 Table).

Stratified analyses

There was no difference in qXR v3 sensitivity when stratified by sex, age, prior TB, HIV, symptoms, or smear status (Fig S1). qXR v3 specificity was higher in people without prior TB, people with cough less than 2 weeks, and who did not report weight loss (Fig S2).

Screening cohort

Since there was only one person with confirmed TB in the screening group (who did have a qXR positive result), we only report specificity. Using the manufacturer’s pre-specified thresholds, the specificity for qXR v3 was 0.93 (95% CI 0.89-0.97) using the culture reference standard and 0.96 (95% CI 0.92-0.98) using the Xpert reference standard (S3 Table).
