# Supplementary material for: Accuracy of digital chest x-ray analysis with artificial intelligence software as a triage and screening tool in hospitalized patients being evaluated for tuberculosis in Lima, Peru
Source: PLOS Glob Public Health. 2024 Feb 7;4(2):e0002031. doi: 10.1371/journal.pgph.0002031 (PMC10849246; doi:10.1371/journal.pgph.0002031)
Supplement: Attachment — Submitted filename: PGPH-D-23-00918 Response to Reviewers_10.20.23.docx [file pgph.0002031.s011.docx]

**PGPH-D-23-00918: Response to reviewers**

**We thank the reviewers for their positive feedback and helpful suggestions. We have addressed all comments, please see responses in bold below and manuscript in tracked changes version (line references below refer to tracked changes version, text in italics is from revised manuscript).**

Reviewer #1: Very well written paper, with an overall sound analysis. Provides needed data on a novel application of cad technology: assessment of people being hospitalized to reduce nosocomial transmission of TB.

I recommend the authors address the following to facilitate reader evaluation of the study, and to strengthen some aspects of the reporting and interpretation to be more consistent with the methods and results.

**We thank the reviewer for their positive feedback and valuable suggestions, which have strengthened our manuscript.**

- This was a cross-sectional study embedded in a larger prospective study. Please add a participant flow diagram that relates the sub-study population to the larger study population of the prospective study.

**We have revised the study flow diagram, Figure 1, to make it clearer how the triage and screening cohorts for the dCXR diagnostic accuracy sub-study relate to the larger FAST implementation study. This diagram illustrates that of the 1006 participants enrolled (with cough and/or TB risk factors) in the larger FAST study during the period of enrollment for the cross-sectional dCXR accuracy study, all 1006 participants were enrolled in the triage cohort of the cross-sectional dCXR accuracy study. We subsequently detail reasons patients were excluded from the diagnostic accuracy study, primarily due to being ineligible for dCXR due to not being admitted through the emergency room, which was one of the requirements stipulated by the study hospital’s radiology department. The screening cohort for the cross-sectional dCXR accuracy study consisted of individuals who were assessed for eligibility for the parent FAST study but were ineligible because they did not have cough or TB risk factors. We explain that the rationale for the screening cohort was to assess the accuracy of dCXR in a lower risk population.**

- Please describe how patients were selected for enrollment into the substudy. Randomly approached, vs consecutively etc

**Participants were consecutively screened for the triage cohort, please see lines 106-107. We clarify in the text that all patients enrolled in the larger FAST study were eligible for this cross-sectional dCXR accuracy study, please see lines 109-110.**

***‘Individuals who were eligible for the parent FAST study were eligible for the triage cohort’***

**The screening cohort consisted of participants who were ineligible for the parent FAST study due to the lack of cough or TB risk factors. As agreed upon with the hospital radiology department to manage workflow, every one in five of these patients were approached for enrollment into the screening cohort, please see lines 118-129.**

- It seems inconsistent that a risk factor that permitted someone to be eligible was 'current active TB diagnosis' but then people on active TB treatment were excluded. Can you please edit the phrasing so that it is no longer inconsistent?

**Patients with an active TB diagnosis were eligible to be enrolled in the larger FAST study and the diagnostic accuracy study, however, as is typical for diagnostic accuracy studies, we excluded patients who were already on TB treatment from the dCXR diagnostic accuracy study. The parent FAST study sought to understand whether patients with a TB diagnosis were on effective treatment and thus did not exclude these patients on the basis of being on TB treatment. Please see lines 112-113:**

***‘a current active TB diagnosis (however patients who were already on TB treatment were subsequently excluded from this diagnostic accuracy sub-study)’***

- If only 2 sputum samples were submitted, 1 for culture and 1 for Xpert, how could some participants have more than one culture result or more than one Xpert (lines 130-132)?

**Participants had at least two sputum specimens collected (see line 141) i.e. some participants provided more than two specimens and thus had more than one culture or Xpert result.**

- A single sputum culture is an imperfect reference standard for pulmonary TB. A single sputum culture is about 73% sensitive for smear-positive TB, and only 61% sensitive for smear-negative TB (see Nelson et al. 1998). A combination of two cultures is over 90% sensitive for smear-negative TB. Similar limitations apply to Xpert. As such, a useful sensitivity analysis would be to assess qxr sensitivity and specificity against a reference standard that includes both TB culture and Xpert (where if either one is positive then the participant is classified as having active TB).

**Thank you for this comment. As recommended, we performed an additional analysis using a combined reference standard which was positive if either mycobacterial culture or Xpert was positive (and negative if both were negative). The sensitivity and specificity of qXR (94% and 36% respectively) against this combined reference were similar to our primary results. These data are included in lines 234-236 and Table S3.**

- Can the authors comment on above in the discussion: do they consider possible source of bias or not?

**We have added text in the discussion, please see lines 368-371 that acknowledges the challenges posed by imperfect reference standards. However, in this study focused on pulmonary TB in a low HIV-prevalence setting, we suspect paucibacillary disease (which may cause not only culture and Xpert to be negative but may also cause CXR to be read as normal) is less likely.**

***‘We acknowledge the challenges posed by imperfect reference standards for TB diagnostic accuracy studies, although we suspect that paucibacillary disease (which could cause culture, Xpert, and also CXR to be negative) is less likely in a hospitalized cohort in a low-HIV prevalence setting.’***

- What was rationale for excluding household contacts from the screening participant cohort? Why not include them in the triage cohort? (line 162-163)

**The rationale for excluding household contacts was that the aim of examining dCXR accuracy in the screening cohort was to see whether and how often people who had low identified risk of having TB actually might have TB disease (based on prevalence survey data from other higher TB incidence settings that demonstrate a large number of people with TB may not have symptoms). Being a household contact constituted TB risk which is why these people were excluded post enrollment from the screening cohort (represents a failure of enrollment).**

**We agree that these participants initially enrolled in the screening cohort who were subsequently identified to be household contacts could have then been subsequently enrolled into the triage cohort, however our team may not have realized this discrepancy between participants denying having TB contacts on the initial screening and subsequently answering yes to the question about being a contact in real time to then enable enrollment of these participants into the triage group.**

- could the authors comment on differences in characteristics between included and excluded participants-- in Results, and perhaps add a supplement table?

**We have added a supplemental table S2. The primary reason for excluding patients from the triage cohort was that they were not admitted through the emergency department (n=397/517), which was required for us to be able to obtain dCXR. This may mean these excluded patients were typically less sick than the included patients in the triage cohort and may account for some of the differences noted in Table S2 (younger, more likely to be female, less likely to be smokers, less likely to be incarcerated or have diabetes, and less likely to have TB symptoms). We added text in the results in lines 208-211 and discussion in line 372-374.**

***The primary reason for excluding patients from the triage cohort was that they were not admitted through the emergency department (n=378/475), which was required for us to be able to obtain dCXR.***

***Limitations of our study are that digital CXR could only be performed on inpatients admitted through the emergency room (which may bias the study towards sicker hospitalized patients).***

- could the authors report Xpert sensitivity for smear-negative culture-positive TB in this cohort?

**We added text in line 198-199 to report ‘*Xpert sensitivity for both smear-positive and negative culture confirmed TB was high (86% and 95%)*, see Table S1b. While Xpert sensitivity for smear negative TB was higher than expected, we note the small sample size for this analysis and wide confidence interval, which includes the results from systematic reviews of Xpert accuracy.**

- was there a reason that alcohol use disorder and diabetes are reported but not evaluated as stratification variables?

**Alcohol use disorder and diabetes have now been added as stratification variables to revised figures 3 and 4.**

- I have major concerns about the interpretation of the stratified analyses for HIV status.
The sample size was small, and likely the stratification was underpowered to detect a difference. The point estimate for sensitivity of qxr for PLHIV is 27% lower. I do not agree with the authors' interpretation that HIV had no impact on sensitivity.

Similarly for specificity, sample size remains an issue. It is surprising that HIV status was associated with increased specificity- can the authors compare their result to other literature in the discussion?

**We thank the reviewer for this comment. We agree that the low sample size of PLHIV limits conclusions about accuracy in this group and have added the following line in the Discussion to address this, see lines 376-379.**

***While dCXR/CAD specificity is typically lower in PWH, in contrast to our findings, we note low numbers in certain subgroups, including the number with HIV due to the low incidence of HIV in Peru and number with smear negative disease, also limit the power to detect differences in our stratified analyses.***

Also can the authors clarify why the point estimate of 0.54 is not equal to 13/22?

**Thank you for identifying this inconsistency. We have double checked and the correct proportion is 15/28 = 0.54, this is now fixed in Figure 4.**

- For smear-negative disease, point estimates are also lower for sensitivity compared to smear positive disease (by 10%). Could this difference have been underestimated due to use of single culture or xpert as reference standard? It is biologically plausible that radiograph will be less sensitive for less extensive disease. This would be something of value to comment on in the discussion, and compare to other studies.

**We have added text in lines 263-266 commenting on these point estimates stratified by smear status. However, similarly to the aforementioned point the reviewer made re: low power to detect differences in the stratified analyses, we are hesitant to emphasize this finding, especially since as the reviewer notes the lower sensitivity for smear negative disease is expected and biologically plausible based on other studies, and have added this to the discussion in lines 376-379.**

***‘Similarly, qXR v4 sensitivity appeared to be higher in smear-positive compared to smear-negative disease, but did not reach statistical significance and we note numbers of participants with smear negative disease were low.’***

***‘..we note low numbers in certain subgroups, including the number with HIV due to the low incidence of HIV in Peru and number with smear negative disease, also limit the power to detect differences in our stratified analyses.’***

- The authors comment on limited precision of their main analysis due to sample size limitations. Would this not also mean some limitations to power to detect differences in stratified analyses? If so- consider adding also as a limitation.

**We agree with this point and have added the following text in limitations, see lines 376-379.**

***‘..we note low numbers in certain subgroups, including the number with HIV due to the low incidence of HIV in Peru and number with smear negative disease, also limit the power to detect differences in our stratified analyses.***’

- The use of liquid culture and Xpert is a strength of the study, most studies in the field only use Xpert as the reference standard. I would recommend the authors add this as a strength.

**Thank you. We have added this in lines 370-372.**

***‘The inclusion of reference standard data from both mycobacterial culture and Xpert is also a strength, since many diagnostic studies only use Xpert as the refence standard.’***

Reviewer #2: The authors have clearly described the methods and results of CAD software evaluation, which shows the software has high sensitivity for TB screening in certain use cases. They have also adequately described the limitations of the sample and yield for the interpretation of software performance in other use cases.

**We thank the reviewer for their positive feedback and valuable suggestions, which have strengthened our manuscript.**

Methods:
The recruitment cascade and participant sample included in the final analysis could be better described. For the triage data set, it is clear – people were enrolled and tested based on symptoms and the majority got a CXR. But it is not clear what the source population is for the screening cohort, nor how they were indicated for TB testing. Was this a subset of the population verbally screened, who had a CXR abnormal result from a radiologist? If this is the case, it would be nice to see the population screened and how it gets split into Cohort 1 & 2 (and their relative sizes). Does the analysis data set include individuals who have CXR normal result from a human reader (live interpretation) and an abnormal result from the CAD software (retrospective interpretation)?

**The rationale behind examining dCXR accuracy in a screening cohort was to see whether and how often people who had low identified risk of having TB i.e. who were not otherwise indicated to have TB testing actually might have TB disease. This research question stems from prevalence survey data from other higher TB incidence settings that demonstrate a large number of people with TB may not have symptoms, so we wanted to determine whether this may be the case in our setting in Lima. We have added text in the Methods to clarify this in lines 114-129 (please also see response to Reviewer 1 and revised study flow diagram, Figure 1). As the reviewer surmises, the source population for the screening population was a subset of the population screened for eligibility into the overall FAST study who did not have cough or TB risk factors and we clarify how we randomly approached every 1 in 5 people in this group for enrollment into the screening cohort. The screening cohort would have otherwise not received CXR so did not have other CXR results other than those we performed for the purposes of this diagnostic accuracy study.**

***‘The rationale for adding a screening cohort to the diagnostic accuracy sub-study was to see the number of patients admitted in our setting in Lima without identified TB risk who may have undiagnosed TB (based on prevalence survey data from other higher TB incidence settings). Every one in five patients with a negative symptom or TB risk screen (undertaken by our FAST implementation study team) was randomly approached for enrollment into the screening cohort for this diagnostic sub-study.’***

**CXR reporting for clinical practice (live interpretation by a human reader) is inconsistently performed in our study setting, with results often handwritten on medical records, if at all, and we were thus unable to undertake a comparison of results from dCXR between human readers and dCXR software. However, we note that prior data on this question including systematic reviews that informed WHO consolidated TB screening guidelines^1^ demonstrate sufficiently similar performance between dCXR software and human readers such that the guidelines state that: ‘computer-aided detection software programmes may be used in place of human readers for interpreting digital chest X-rays for screening and triage for TB disease’.**

Results/Discussion:
The low yield in the screening cohort is interesting (NNT almost 200). We need to understand how this population was recruited in order to better understand their risk (see comment above). The discussion notes that contacts and people with past history of TB are excluded, maybe making this population lower-risk. But if they were primarily symptom negative, CXR abnormal (by a radiologist), it is also possible that the radiologist has issues with CXR interpretation quality.

**Thank you for this comment. We have clarified in the response above and in the revised manuscript, see lines 114-129 how the screening group were recruited and clarified that the aim was that this group would be low-risk for TB (i.e. without identified risk) and have clarified that they did not have a priori abnormal CXRs. We also note that the reference standard for our study was microbiological or molecular test confirmation, and hence CXR interpretation quality as part of the TB diagnostic process was not a concern here.**

The conclusion of low specificity is accurate, but the discussion (and final conclusions paragraph) doesn’t sufficiently put this finding into context. The low specificity calculation is among study participants who are heavily pre-screened and have a microbiological result, not among hospital clients who are screened via FAST. In the context of FAST, True Negative people are likely to be correctly triaged by the symptoms and CXR screens and to never get the opportunity to receive a microbiological test to confirm their True Negative status. Normal CXR images are easier to discerning whether an abnormality is TB related or not. So the evaluation’s low specificity finding is very likely to be underestimating the CAD software’s performance among the entire screened population. If there was a composite reference standard where CXR normal FAST participants were considered a True Negative – or even better a double CXR normal result – you’d likely see significantly higher specificity. Text acknowledging this in the discussion – or at least that this population as not included in your specificity calculations – should be added to the discussion.

**Thank you for this comment. The reviewer is correct to point out that test performance depends on context. Population prevalence will affect the predictive value of the CAD software testing. We have added text in the discussion in lines 336-341 to reflect that the triage cohort represents a highly screened cohort with a higher pre-test probability of TB or lung disease and that negative predictive value for this CAD software depends on population disease prevalence.**

***‘We also note that this diagnostic accuracy assessment in the triage cohort reflects use of the test in a pre-screened population who had a high pre-test probability of TB or other lung disease and underwent microbiological testing that revealed a high prevalence of TB. Thus negative predictive value would be lower for this cohort than if qXR testing was applied to the population of people initially screened (rather than those enrolled) for FAST.’***

**References**

1. World Health Organization. Consolidated guidelines on tuberculosis, Module 2: Screening, Systematic screening for tuberculosis disease. Published online 2021. https://reliefweb.int/report/world/who-consolidated-guidelines-tuberculosis-module-2-screening-systematic-screening
