# Supplementary material for: Accuracy of digital chest x-ray analysis with artificial intelligence software as a triage and screening tool in hospitalized patients being evaluated for tuberculosis in Lima, Peru
Source: PLOS Glob Public Health. 2024 Feb 7;4(2):e0002031. doi: 10.1371/journal.pgph.0002031 (PMC10849246; doi:10.1371/journal.pgph.0002031)
Supplement: Attachment — Submitted filename: PGPH-D-23-00918 Response to Reviewers_11.28.23.docx [file pgph.0002031.s012.docx]

**PGPH-D-23-00918 R2: Response to reviewers**

**We thank the editor and reviewers for their thorough re-review and helpful suggestion to remove the sentence about the stratified analyses from the abstract and clarify the associated sentence in the discussion, given these analyses were underpowered.**

**As an additional strength, we have also now updated the analyses with the latest version of qXR v4 (now final CE-marked version). This resulted in minor changes to qXR diagnostic accuracy results which have now been updated in the manuscript (noted in tracked changes version) but has not changed the overall findings for either the triage or screening cohorts.**

**We have addressed all comments, please see responses in bold below and manuscript (line references below refer to tracked changes version, text in italics is from revised manuscript).**

Editor comment (please see response to this and linked reviewer comment below):

The comments from the review have overall been addressed; the first reviewer has made an additional comment related to the abstract. I would consider their suggestion, or removing the sentence or clarifying the sentence so that the stratified results are described but not compared given that it is underpowered.

Reviewer #1: The authors have adequately addressed my concerns. However, I strongly suggest a change to the abstract which would be in keeping with the authors' own comments about power to detect differences in stratified analyses-- which was low. The following cannot be concluded due to the low power: "qXR sensitivity did not differ stratified by sex, age, prior TB, HIV, and symptoms."

Please remove that sentence and replace with a statement that the study was underpowered to detect meaningful differences in sensitivity stratified by sex, age, prior TB, smear status, HIV status, and symptoms.

**We thank the reviewer for this comment and agree with the reservation about including the results from the stratified analyses in the abstract. For clarity, we have removed this sentence as suggested by the editor, and instead replaced it with a sentence (lines 37-39) describing the high prevalence of lung abnormalities detected by the software in the triage group, as this is of relevance given low specificity in our hospitalized cohort.**

***A high prevalence of radiographic lung abnormalities, most notably opacities (81%), consolidation (62%), or nodules (58%), was detected by qXR on digital CXR images from the triage cohort.***

Minor comment:
The sentence is difficult to follow- can it be clarified? "While dCXR/CAD specificity is typically lower in PWH, in contrast to our findings, we note low numbers in certain subgroups, including the number with HIV due to the low incidence of HIV in Peru and number with smear negative disease, also limit the power to detect differences in our stratified analyses."

**Thank you. We shortened this statement in lines 353-355 for clarity, which is aligned with the reviewer’s prior comments about not overinterpreting results from the stratified analyses given they were underpowered.**

***We note low numbers in certain subgroups, including the number with HIV due to the low incidence of HIV in Peru and number with smear negative disease, also limit the power to detect differences in our stratified analyses.***

Reviewer #2: All comments have been fully addressed.

**Thank you.**
